# Anisotropic transverse magnetoresistance and Fermi surface in TaSb_2_

**DOI:** 10.1038/s41598-018-28922-9

**Published:** 2018-07-12

**Authors:** Arnab Pariari, Ratnadwip Singha, Shubhankar Roy, Biswarup Satpati, Prabhat Mandal

**Affiliations:** Saha Institute of Nuclear Physics, HBNI, 1/AF Bidhannagar, Kolkata, 700 064 India

## Abstract

TaSb_2_ has been predicted theoretically to be a weak topological insulator. Whereas, the earlier magnetotransport experiment has established it as a topological semimetal. In the previous works, the Shubnikov-de Haas oscillation has been analyzed to probe the Fermi surface, with magnetic field along a particular crystallographic axis only. By employing a sample rotator, we reveal highly anisotropic transverse magnetoresistance by rotating the magnetic field along different crystallographic directions. To probe the anisotropy in the Fermi surface, we have performed magnetization measurements and detected strong de Haas-van Alphen (dHvA) oscillations for the magnetic field applied along *a* and *b* axes as well as perpendicular to *ab* plane of the crystals. Three Fermi pockets have been identified by analyzing the dHvA oscillations. With the application of magnetic field along different crystal directions, the cross-sectional areas of the Fermi pockets have been found significantly different, i.e., the Fermi pockets are highly anisotropic in nature. Three-band fitting of electrical and Hall conductivity reveals two high mobility electron pockets and one low mobility hole pocket. The angular variation of transverse magnetoresistance has been qualitatively explained using the results of dHvA oscillations and three-band analysis.

## Introduction

Inclusion of topology in electronic band structure has opened-up a new era in condensed matter research^1,2^. Three-dimensional (3D) Dirac and Weyl materials are the most recent discovery on the topological phases of matter, described as topological semimetal^3–10^. Unlike topological insulators, these systems host semimetallic bulk with linearly dispersing excitation and their surface state is topology protected Fermi arc. Due to the unique band topology, they show several exotic electronic properties of technological and fundamental interest, such as ultra-high carrier mobility (*μ*), giant magnetoresistance (MR), chiral anomaly and Berry phase induced anomalous Hall effect^11^, unlike conventional metal and semimetals. On the other hand, the nature and geometry of the Fermi surface can also modify the electronic transport significantly in a material. Such as, the anisotropic magnetoresistance has been ascribed to the anisotropy of the Fermi surface^12–14^. Thus, it is important to acquire the knowledge of Fermi surface to explain different electronic properties of a material.

Without taking into account the role of spin-orbit coupling, TaSb_2_ has been proposed to be a topological semimetal. Upon inclusion of spin-orbit coupling, however, gap opens-up at each band crossing point^15–18^. This leads to the possibility of suppressed linear electronic dispersion in TaSb_2_. On the other hand, the transport experiments have established the 3D Dirac fermionic excitation through the observation of negative longitudinal magnetoresistance (LMR) and detection of non-trivial *π* Berry’s phase in Landau level index plot^15,18^. So, every further investigation is complementary to the previous results on the electronic band topology of TaSb_2_. Apart from this unconventional nature of electronic band structure, large magnetoresistance and the presence of two or three Fermi pockets depending on the position of the Fermi level, have been reported in earlier works by analysing the Shubnikov-de Haas (SdH) oscillation for magnetic field along one of the crystallographic axes^15,17,18^. Due to the lower symmetry (monoclinic) of crystal structure of TaSb_2_, one expects a strong anisotropy in Fermi surface of this system. However, the crystallographic direction dependence of magnetotransport properties and the anisotropy of the Fermi surfaces have not been probed so far. This is important not only for understanding different electronic properties controlled by the Fermi surface but also helpful for the application point of view. In the present work, we have shown large anisotropy in magnetoresistance when the magnetic field is applied along different crystallographic directions in transverse experimental configuration (field is perpendicular to the current direction). Besides this, employing magnetization measurements along three mutually perpendicular directions on the same single crystal and by analysing the de Haas-van Alphen (dHvA) oscillation, we report the anisotropic nature of the Fermi pockets.

## Results

### Outcomes of High resolution transmission electron microscopy (HRTEM) and Powdered x-ray diffraction

**Figure 1:**
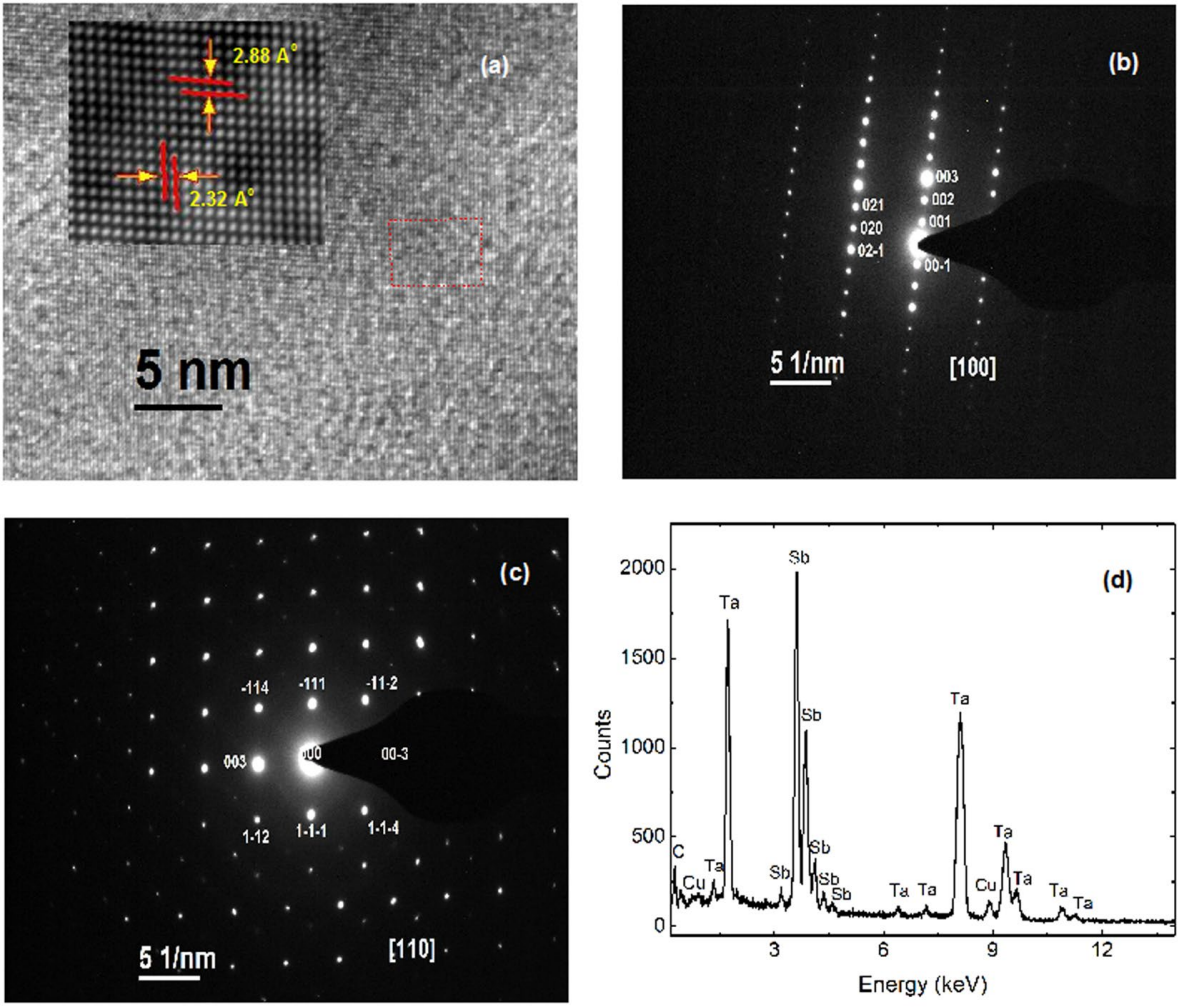
(**a**) High resolution TEM image, taken on a representative piece of TaSb_2_ single crystal. Inset shows the Fourier-filtered image of the red dotted region. (**b**) and (**c**) are the selected area electron diffraction (SAD) patterns taken along [100] and [110] zone axis, respectively. (**d**) The energy-dispersive X-ray (EDX) spectroscopy data.

**Figure 2:**
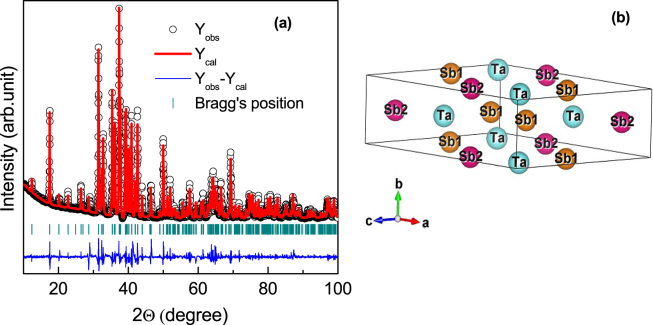
(**a**) X-ray diffraction pattern of powdered single crystals of TaSb_2_. Black open circles are experimental data (Y_*obs*_), red line is the calculated pattern (Y_*cal*_), blue line is the difference between experimental and calculated intensities (Y_*obs*_ − Y_*cal*_), and green lines show the Bragg positions. (**b**) Schematic diagram for the crystal structure of TaSb_2_.

The HRTEM image of a representative piece of sample, which has been taken from a single crystal of TaSb_2_ is shown in Fig. 1(a). Very clear periodic lattice structure implies that there is no secondary phase or atom clustering or disorder in the present sample. The Fourier-filtered image of the selected region in the inset, shows inter-planar spacings (*d*-spacing) of 2.88 Å and 2.32 Å. These measured *d*-spacings are close to the (111), and (003) inter-planar spacings of TaSb_2_ (JCPDS # 65-7656). Figure 1(b,c) show the selected area electron diffraction (SAD) pattern recorded along [100] and [110] zone axis, respectively. The periodic pattern of the spots in SAD implies high-quality single crystalline nature of the grown samples. The diffraction pattern was indexed using the lattice parameters of monoclinic TaSb_2_. The energy-dispersive x-ray (EDX) spectrum, as shown in Fig. 1(d), confirms the presence of the elements in desired stoichiometry. Please note that the carbon and copper peaks in spectrum appear from the carbon coated copper grid on which the sample was mounted for TEM analysis. Fig. 2(a) shows the high-resolution x-ray diffraction pattern of the powdered sample of TaSb_2_ crystals at room temperature. Within the resolution of XRD, we did not see any peak due to the impurity phase. Using the Rietveld profile refinement, we have calculated the lattice parameters *a* = 10.221, *b* = 3.645 and *c* = 8.291 Å, and *β* = 120.40° with space-group symmetry *C*_12/*m*1_. A sketch map of the crystal structure of TaSb_2_ has been shown in Fig. 2(b).

### Temperature dependence of resistivity both in absence and presence of external magnetic field

**Figure 3:**
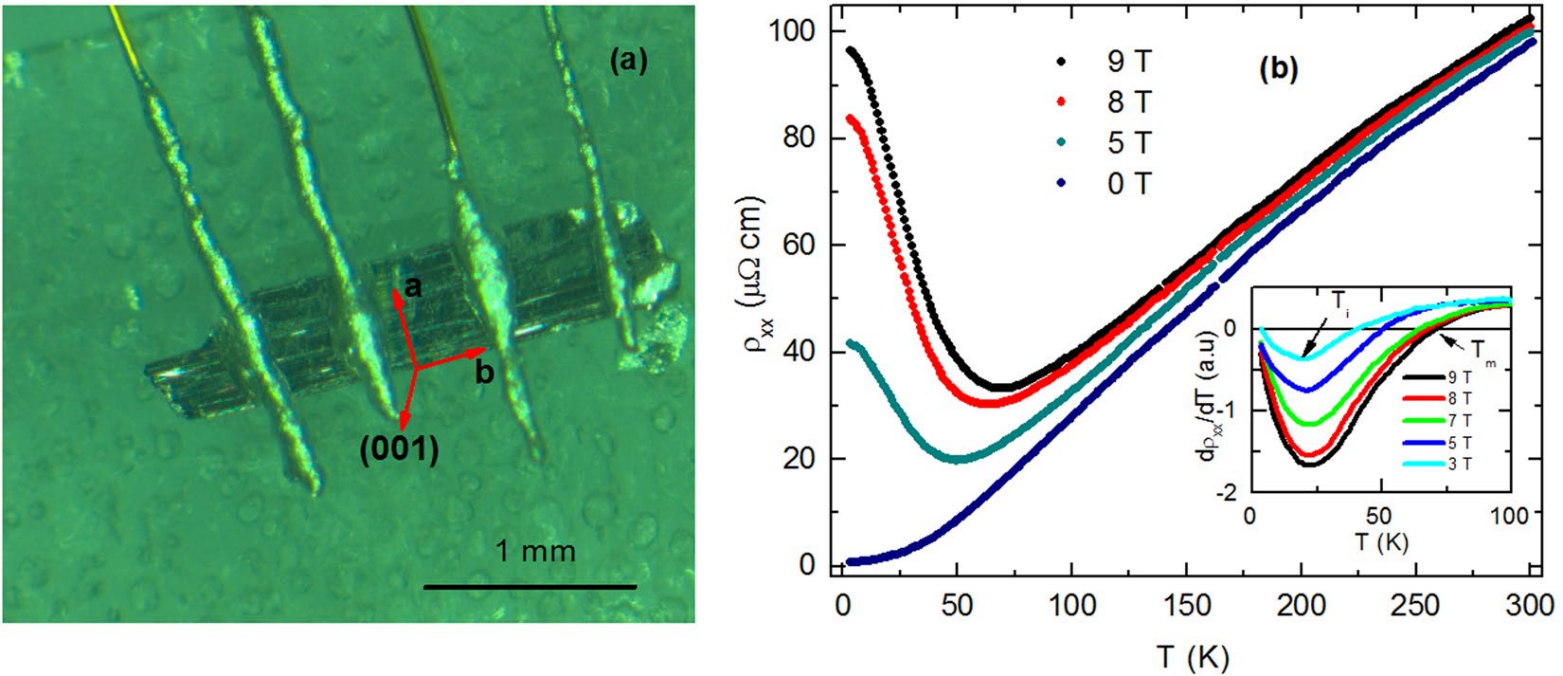
(**a**) Typical morphology and different crystallographic directions of a representative single crystal of TaSb_2_, and (**b**) Temperature dependence of resistivity (*ρ*_*xx*_) both in presence and absence of external magnetic field. Current (*I*) is applied along **b**-axis and magnetic field (*B*) is perpendicular to the **ab** plane. Inset shows the first order derivative of *ρ*_*xx*_ with respect to *T*. Metal- to semiconductor-like crossover temperature is named as *T*_*m*_, and *T*_*i*_ is the temperature for the inflection point of *ρ*_*xx*_(*T*).

Figure 3(a) shows a representative single crystal of TaSb_2_ with four electrical contacts. The typical length of the single crystals is ∼2 mm. This type of material, known as transition metal dipnictide MPn_2_ [M = V, Nb, Ta, Cr, Mo, and W, Pn = P, As, and Sb], grows preferentially along **b**-axis. As a consequence, the longer direction of the crystal is the **b**-axis^12,19^. The crystallographic **a**-axis is perpendicular to **b**-axis, and both the axes lie on the largest flat plane of the crystal. Because of the monoclinic structure of the material, crystallographic **c**-axis is not perpendicular to **ab** plane. For convenience, we have considered three mutually perpendicular directions on the crystal as reference. Two of them are the crystallographic **a**- and **b**-axis, and the third one is perpendicular to **ab** plane, i.e., (001) direction. The zero-field resistivity (*ρ*_*xx*_) is metallic over the whole temperature range, as shown in Fig. 3(b). *ρ*_*xx*_ shows strong *T* dependence. Small value of *ρ*_*xx*_ at 2 K (∼0.75 *μ*Ω cm) and the large residual resistivity ratio, *ρ*_*xx*_(300 K)/*ρ*_*xx*_(2 K) ∼ 130, indicate good quality of the single crystals. With the application of magnetic field, the low-temperature resistivity drastically enhances. As a result, a metal- to semiconductor-like crossover behavior starts to appear with decreasing temperature. With the increase in field strength, the semiconducting-like behavior becomes more and more prominent, and the metal- to semiconductor-like crossover temperature (*T*_*m*_) shifts towards higher temperature side, as evident from the inset of Fig. 3(b). Unlike *T*_*m*_, the temperature (*T*_*i*_) at which *dρ*_*xx*_/*dT* exhibits a minimum is almost independent of the strength of the magnetic field and remains fixed at ∼20 K. Slightly below *T*_*i*_, the saturation-like behavior in *ρ*_*xx*_(*T*) starts to appear. The magnetic field induced metal-semiconductor crossover and the low-temperature resistivity plateau are the common phenomena in topological semimetals^15,17,20–24^. Different explanations such as magnetic field induced gap opening at the Dirac node^21,24^ and Kohler’s scaling of magnetoresistance^25^ have been proposed as possible origins.

### Angular dependence of magnetoresistance

**Figure 4:**
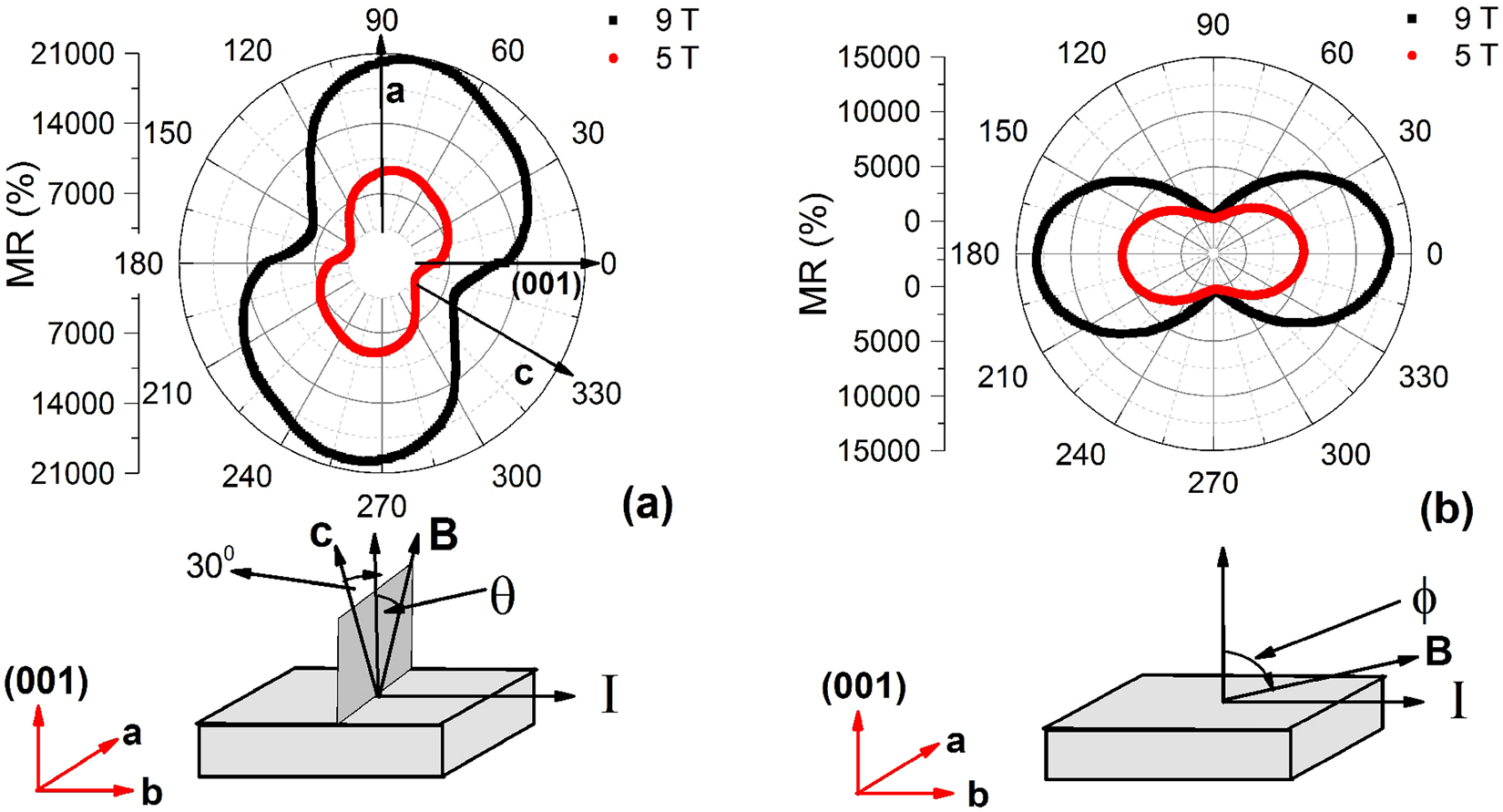
(**a**) Anisotropic magnetoresistance of a representative TaSb_2_ single crystal at 2 K when the direction of magnetic field changes from (001) to **a**-axis, making an angle *θ*. (**b**) Magnetoresistance of the same piece of single crystal at 2 K when the angle (90° − *ϕ*) between *I* and *B* has been changed at two representative field strengths 5 and 9 T.

Several topological semimetals like NbSb_2_, ZrSiS and TaAs_2_ show anisotropic magnetoresistance with respect to the field direction, which arises due to the anisotropy in their Fermi surface^12,14,19,26–29^. This anisotropic magnetoresistance has significant impact in technological application and device fabrication. Fig. 4(a) shows the transverse magnetoresistance (*B* ⊥ *I*) for the TaSb_2_ single crystal at 2 K with the rotation of field about **b**-axis. When the field is along the (001) direction, the MR, which is defined as [*ρ*_*xx*_(*B*) − *ρ*_*xx*_(0)]/*ρ*_*xx*_(0), is ∼1.3 × 10^4^% at 9 T and ∼4 × 10^3^% at 5 T. As the direction of the field is changed from (001) towards **a** direction, the value of MR is observed to increase and becomes maximum (∼2 × 10 ^4^% at 9 T) at around *θ* = 75°. MR is minimum ∼9500% at around 165°. The polar plot in Fig. 4(a) shows a two-fold rotational symmetry, which is consistent with the monoclinic crystal structure of the present sample. The tilted pattern of MR(*θ*) with respect to the crystallographic axis may be due to the complex geometry of the Fermi surfaces and their relative contribution to transport^19,30^.

Figure 4(b) shows the typical behavior of MR at 2 K when the angle (90° − *ϕ*) between *I* and *B* has been varied continuously. As expected, due to the orbital origin of MR, the maximum and minimum in MR appear at *ϕ* = 0° and *ϕ* = 90°, respectively. This also confirms that there is no intrinsic misalignment between *I* and *B* in our crystal. Within the resolution of the angular variation of horizontal sample rotator, we have not observed any detectable negative MR under $B\parallel I$ configuration, i.e., in longitudinal set up. To further verify, we have also measured the field dependence of MR at small angle interval (∼1°) around *ϕ* = 90°. The field dependence of MR at *ϕ* = 90° configuration has been shown and discussed in the Supplementary Information (see Fig. S1). We have repeated the same experiment on other single crystals but failed to detect any negative MR for *ϕ* close to 90°. This implies that the negative longitudinal MR (NLMR) is either absent or weak in competition with unavoidable misalignment induced positive MR in the present compound. In this context, it is worthy to mention that the NLMR has been reported in isostructural compounds TaAs_2_ and NbAs_2_ by Li *et al*.^18^. But, the subsequent work has established that the NLMR in TaAs_2_ and NbAs_2_ is due to the inhomogeneous current distribution inside the sample, i.e., due to the current jetting effect^19^. The NLMR disappears when the electrical contacts are made correctly^19^.

### Field dependence of MR for *B* || (001) and *I* || b, and for B || a and I || b configurations

**Figure 5:**
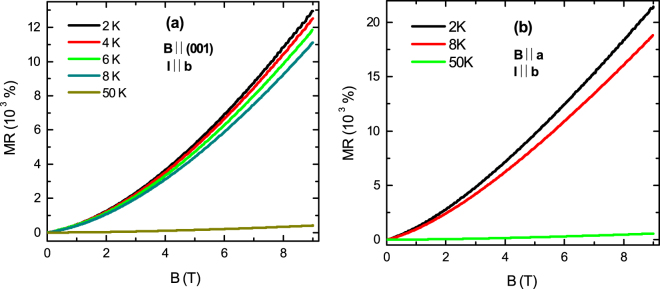
(**a**) Magnetoresistance as a function of magnetic field at some representative temperatures for (**a**) *B* || (001) and *I* || **b** configuration, and (**b**) for *B* || **a** and *I* || **b** configuration.

Figure 5(a) shows the field dependence of MR for *B* || (001) and *I* || **b** configuration. The large non-saturating MR up to 9 T suppresses with increasing temperature. Over the entire field range, it shows approximately ∼*B*^1.5^ dependence. Below 5 K, a high frequency Shubnikov-de Haas effect has been observed in the high-field region. Due to very small amplitude of the oscillation, it is difficult to extract the oscillating part from the large polynomial background for calculating any physical parameter accurately. The amplitude of oscillation also suppresses very rapidly with increasing temperature. So, we have employed the de Haas-van Alphen oscillation in magnetization measurements to probe the Fermi surface. Figure 5(b) shows MR *vs B* for *B* || **a** and *I* || **b** configuration. The value of MR is larger in this direction as evident from Fig. 4(a), and suppresses rapidly with increasing temperature.

### Hall measurements and multi-band analysis

**Figure 6:**
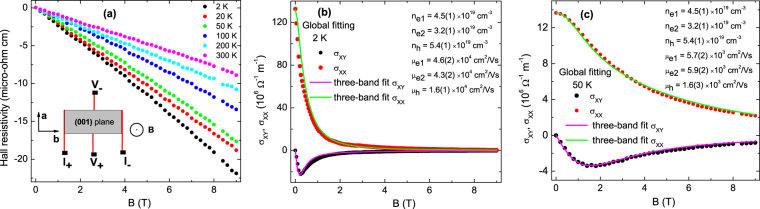
(**a**) The field dependence of Hall resistivity (*ρ*_*yx*_) and its evolution with temperature. The Hall measurement has been performed in *B* || (001) and *I* || **b** configuration. Inset shows the schematic of the Hall measurement setup. (**b**) and (**c**) show the simultaneous (i.e., global) three-band analysis of electrical conductivity (*σ*_*xx*_) and Hall conductivity (*σ*_*xy*_) data at 2 K and 50 K. The black dots represent the field dependence of Hall conductivity (*σ*_*xy*_) and the red dots represent the field dependence of electrical conductivity (*σ*_*xx*_). The green and magenta curves are the three-band fit to the experimental data with the expression, *σ*_*xx*_ = ${\sum }_{i=1}^{3}\frac{e{n}_{i}{\mu }_{i}}{1+{\mu }_{i}^{2}{B}^{2}}$ and *σ*_*xy*_ = ${\sum }_{i=1}^{3}{S}_{i}\frac{e{n}_{i}{\mu }_{i}^{2}B}{1+{\mu }_{i}^{2}{B}^{2}}$, respectively. The density and mobility for each types of charge carrier have been mentioned in the inset.

We have also measured the field dependence of Hall resistivity (*ρ*_*yx*_), and this has been shown in Fig. 6(a). Over the whole temperature and field range *ρ*_*yx*_ is negative and exhibits weak superlinear *B* dependence. This indicates the presence of more than one types of charge carriers and electron dominated transport in TaSb_2_. As three Fermi pockets have been detected in the present de Haas-van Alphen oscillation, which will be discussed in the following sections, we have performed three-band analysis of the electrical conductivity (*σ*_*xx*_) and Hall conductivity (*σ*_*xy*_) to determine *μ* and carrier density (*n*) of the individual Fermi pockets. At first, the *σ*_*xx*_(*B*) = $\frac{{\rho }_{xx}}{{\rho }_{xx}^{2}+{\rho }_{yx}^{2}}$ and *σ*_*xy*_(*B*) = $\frac{{\rho }_{yx}}{{\rho }_{xx}^{2}+{\rho }_{yx}^{2}}$ have been determined, using the experimental *ρ*_*yx*_(*B*) and *ρ*_*xx*_(*B*)^31^. The field dependence of *σ*_*xy*_ in a system of *N* carrier species is given by the expression, *σ*_*xy*_(*B*) = ${\sum }_{i=1}^{N}{S}_{i}\frac{e{n}_{i}{\mu }_{i}^{2}B}{1+{\mu }_{i}^{2}{B}^{2}}$, where *S*_*i*_ is +1 for holes and −1 for electrons^32^. The analogous expression for electrical conductivity is *σ*_*xx*_(*B*) = ${\sum }_{i=1}^{N}\frac{e{n}_{i}{\mu }_{i}}{1+{\mu }_{i}^{2}{B}^{2}}$. Figure 6(b,c) represent the simultaneous three-band fit to the electrical conductivity (*σ*_*xx*_) and Hall conductivity (*σ*_*xy*_) data at representative temperatures 2 K and 50 K. This type of fitting, which is also known as global fitting, includes parameter sharing between the two expressions to obtain the best fit solutions. Analysis reveals two electron-type Fermi pockets with carrier density ∼4.5(1) and 3.2(1) × 10^19^ cm ^−3^, and one hole-type Fermi pocket with *n *∼ 5.4(1) × 10^19^ cm ^−3^. It has been found that the mobility of hole-type carrier (∼1.6 × 10^4^ cm^2^/Vs) is much smaller compared to the electrons from the smaller Fermi pockets. The mobility of electrons from the Fermi pockets of intermediate and smallest volume are ∼4.6(2) and 4.3(2) × 10^4^ cm^2^/Vs, respectively. Three-band fit has also been performed at different other temperatures. This has been shown for 100 and 200 K in Fig. S2. The extracted values of parameters, which have been mentioned in the inset of Fig. S2, are consistent with the above results. This coexistence of electron- and hole-type charge carriers supports the earlier experimental and theoretical works on TaSb_2_^16–18^. The present result also supports the electron-hole compensation mechanism as a possible origin for large and non-saturating magnetoresistance in this family of materials (MPn_2_)^15,19,33,34^.

### Probing the Fermi surface through de Haas-van Alphen oscillation

**Figure 7:**
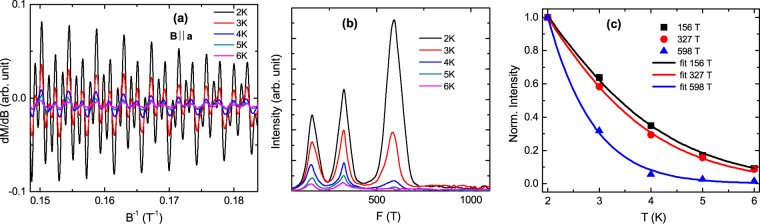
(**a**) Oscillating part of dc susceptibility (Δ*χ*), which has been obtained by taking the first order derivative of magnetization (*M*), as a function of 1/*B* for *B* || **a**-axis configuration. (**b**) The oscillation frequencies after the fast Fourier transformation (FFT). Oscillations in the field range 4 to 7 T has been used for FFT. (**c**) Temperature dependence of the normalized FFT peak intensity. The solid line is a fit to the Lifshitz-Kosevich formula [equation (1)].

To probe the Fermi surface of TaSb_2_, we measured the field dependence of magnetization (*M*) of the representative piece of single crystal, and observed prominent de Haas-van Alphen oscillations within 7 T magnetic fields (Fig. S3). Taking the first order derivative of *M* with respect to *B*, the oscillating component of the susceptibility has been obtained and shown in the Fig. 7(a) for *B* || **a** configuration. From the figure, it is evident that the oscillation amplitude rapidly suppresses with increasing temperature and above 6 K, the amplitude is too small to detect within the experimental field range. The fast Fourier transform (FFT) spectrum in Fig. 7(b) shows three distinct oscillation frequencies (*F*) at 156, 327 and 598 T. Oscillations in the field range 4 to 7 T has been used for FFT in all experimental configurations. Below 4 T, the dHvA oscillation amplitude is too weak to analyze accurately. The obtained values of frequency imply that there are three Fermi pockets in the present sample, similar to that observed in earlier SdH oscillation measurement, where the frequency peaks have been reported at 55, 234 and 487 T^17^. On the other hand, Li *et al*. have observed only two frequency peaks at 220 and 465 T in their SdH oscillation study^15^. Although there are considerable differences between the experimental findings, all of them appears to be allowed for TaSb_2_ crystals. This is due to the fact that TaSb_2_ hosts multiple electronic bands close to the Fermi level and it is not a perfectly compensated semimetal^16^. As a consequence, the number of Fermi pockets and its volume can vary from sample to sample, depending on the strength of doping during preparation. The elaborated discussion is given in Supplementary Information. Employing the Onsager relation *F* = (*ϕ*_0_/2*π*^2^)*A*_*F*_, the cross-sectional areas of the Fermi surfaces normal to the field direction have been calculated and listed in Table 1. A significant difference in *A*_*F*_ of the Fermi pockets has been observed along this direction. The damping of the oscillation amplitude (FFT peak intensity) with temperature can be described by the thermal damping term of the Lifshitz-Kosevich formula:

1 $${\rm{\Delta }}{R}_{T}=a\frac{2{\pi }^{2}{k}_{B}T/\hslash {\omega }_{c}}{\sinh \,\mathrm{(2}{\pi }^{2}{k}_{B}T/\hslash {\omega }_{c})},$$

where *a* is a temperature-independent constant and *ω*_*c*_ is the cyclotron frequency. Figure 7(c) shows the fitting of the FFT peak intensity as a function of temperature with equation (1). Using the extracted value of *ω*_*c*_ from the fitting, the effective cyclotron mass of the charge carrier (*m*_*eff*_) and the Fermi velocity (*v*_*F*_) are obtained from the relations *ω*_*c*_ = *eB*/*m*_*eff*_ and *v*_*F*_ = ℏ*k*_*F*_/*m*_*eff*_, respectively. The calculated parameters are shown in Table 1. The effective mass of the charge carrier for all the three Fermi pockets are smaller than the rest mass of free electron and similar to that reported in earlier SdH oscillation study^17^. Considering spherical approximation with frequency 598 T along all the momentum directions, the over-estimated value of carrier density for the largest Fermi pocket has been found to be ∼8 × 10^19^ cm^−3^, using the expression, ${\rm{\Delta }}(\frac{1}{B})=\frac{2e}{\hslash }{(\frac{{g}_{s}{g}_{v}}{6{\pi }^{2}{n}_{3D}})}^{\mathrm{2/3}}$^35^. Here, ${\rm{\Delta }}(\frac{1}{B})$, g _*s*_, and g _*v*_ are the period of the oscillation, spin degeneracy, and valley degeneracy of the Fermi pockets, respectively. This value of carrier density from the quantum oscillation study is comparable with the value obtained from three-band analysis. The above comparison for a representative Fermi pocket supports the reliability of the values of parameters, determined by the three-band fitting.

**Table 1:** Parameters associated to the Fermi surface of TaSb_2_, when the field is applied along the **a**-axis. *A*_*F*_ is the Fermi surface cross-section. *m*_*eff*_ is the effective mass of the charge carrier and *v*_*F*_ is the Fermi velocity.

Frequency	*A*_*F*_	*meff*	*vF*
T	10^−3 ^Å^−2^	*m*_0_	10^5^ m/s
156(2)	14.8	0.32(1)	2.5
327(2)	31.1	0.35(1)	3.3
598(3)	56.8	0.59(1)	2.6

**Figure 8:**
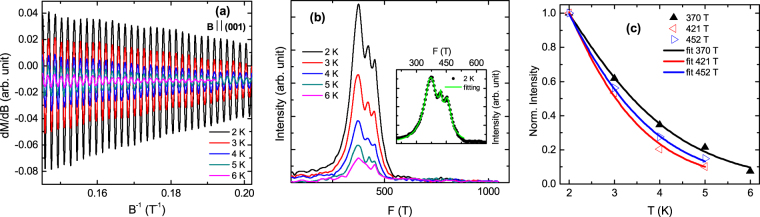
(**a**) Δχ versus 1/*B* for *B* || (001) configuration. (**b**) The oscillation frequencies after the fast Fourier transformation. Oscillations in the field range 4 to 7 T has been used for FFT. A specimen of the theoretical fitting to the frequency distribution plots has been shown in the inset at 2 K. (**c**) Thermal damping of the normalized FFT peak intensity. The solid line is a fit to the Lifshitz-Kosevich formula [equation (1)].

**Figure 9:**
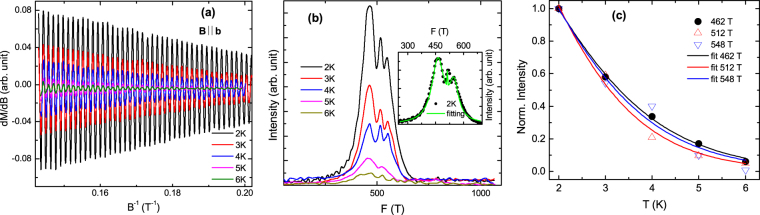
(**a**) Δχ versus 1/*B* for *B* || **b**-axis configuration. (**b**) The oscillation frequencies after the fast Fourier transformation. Oscillations in the field range 4 to 7 T has been used for FFT. A specimen of the theoretical fitting to the frequency distribution plots has been shown in the inset at 2 K. (**c**) Thermal damping of the normalized FFT peak intensity. The solid line is a fit to the Lifshitz-Kosevich formula [equation (1)].

The results of magnetization measurement and the details of dHvA oscillation analysis for the field along (001) direction of the same piece of TaSb_2_ single crystal, have been demonstrated in Fig. 8(a–c). Employing the fast Fourier transformation of the oscillations in Fig. 8(a), three closely spaced frequency peaks 370, 421 and 452 T have been obtained [Fig. 8(b)]. As these frequency peaks are not well resolved, there will be significant modification in the peak intensities due to overlap of three frequency distribution curves. So, it will not be wise to calculate *m*_*eff*_, using these intensities of the peaks. To deal with the situation, we have performed extensive theoretical fitting to the intensity vs frequency curves. The frequency distribution of quantum oscillation has been found to be Lorentzian in nature^35^. So, the superposition of three Lorentzian distribution functions has been used to fit the experimental data (see the Supplementary Information for details). Good quality of the theoretical fit to the frequency distribution plot is evident from the inset of Fig. 8(b) at a representative temperature 2 K. The fittings for other temperatures are shown in Fig. S4 of Supplementary Information. The positions of the peaks, determined from the fitting, have been found to be slightly different from the apparent peak positions in Fig. 8(b) and listed in Table 2. The calculated values of *A*_*F*_ reveal nearly equal cross-sectional area of Fermi pockets, unlike to that observed in *B* || **a**. If we compare the values of *A*_*F*_, the smallest one is ∼140% and the medium one is ∼30% higher in *B* || (001) than the corresponding smallest and medium ones in *B* || **a**, respectively. Whereas, the largest cross-sectional area in *B* || (001) configuration is 25% smaller compared to its counterpart in *B* || **a**. The intensity of the FFT peaks, deduced from the fittings, have been plotted as a function of temperature in Fig. 8(c). Employing the thermal damping term of the Lifshitz-Kosevich formula, we have calculated *m*_*eff*_ and *v*_*F*_ for all the Fermi pockets and listed in Table 2. The values of *m*_*eff*_ in *B* || (001) for the two lighter Fermi pockets are nearly equal to that observed in *B* || **a**, and the massive one (also the largest one) is only 30% less in the previous configuration compared to later. Similar magnetic measurements and dHvA oscillation analysis have been done for the field along **b** crystallographic direction and shown in Fig. 9(a–c). Three Fermi pockets of equivalent cross-sectional area have also been found in this configuration. The values of *A*_*F*_, *m*_*eff*_ and *v*_*F*_ for all the frequencies are presented in Table 3. The values of *m*_*eff*_ are close to each other and comparable to that observed in *B* || (001). Theoretical fit to the thermal damping of FFT peak intensity has been found to have maximum deviation for 548 T frequency. Even in this case, the standard deviation in the calculated value of *m*_*eff*_ is ∼ ±0.02 *m*_0_.

**Table 2:** Parameters associated to the Fermi surface of TaSb_2_, when the field is applied perpendicular to ab plane i.e., along the (001) direction.

Frequency	*A*_*F*_	*meff*	*vF*
T	10^−3^ Å^−2^	*m*_0_	10^5^ m/s
370(2)	35.2	0.32(1)	3.9
421(4)	40.1	0.37(1)	3.5
452(3)	43.0	0.41(1)	3.4

**Table 3:** Parameters associated to the Fermi surface of TaSb_2_, when the field is applied along the **b**-axis direction.

Frequency	*A*_*F*_	*meff*	*vF*
T	10^−3^ Å^−2^	*m*_0_	10^5^ m/s
462(3)	44.0	0.34(1)	4.0
512(5)	48.6	0.39(1)	3.7
548(4)	52.1	0.35(2)	4.2

## Discussions

Transverse magnetoresistance of orbital origin has been shown to scale with the mobility of charge carriers in the plane perpendicular to the applied *B*^30,36^. For an example, the typical field dependence of electrical conductivity is given by *σ*(*B*) $\sim \frac{ne\mu }{1+{\mu }^{2}{B}^{2}}$^30,36,37^. As mobility is a tensor quantity in *B*, the anisotropic behavior of magnetoresistance can be explained by the anisotropy in the mobility tensor^36^. The following discussion on angular dependence of MR in terms of anisotropy in Fermi surfaces is based on the assumption ‘each pocket provides only one dHvA frequency and the dHvA frequencies do not interchange among the three pockets when the field direction is changed’. The mobility of charge carrier in a material is determined by the ratio of the scattering time (*τ*) to the *m*_*eff*_, *μ* ∼ $\frac{\tau }{{m}_{eff}}$. From the analysis of dHvA oscillation, we have seen that two Fermi pockets of small and intermediate volume have much larger cross-section for *B* along (001) direction, i.e., perpendicular to ab plane, compared to the **a**-axis. This implies smaller phase space for the scattering of charge carrier from the two Fermi pockets in the plane perpendicular to **a**-axis under application of *B*, and as a result, the value of *τ* is larger^36^. Whereas, *m*_*eff*_ of charge carriers in these pockets for the above two directions are almost equal. So, the anisotropy in the scattering time of charge carrier from these two Fermi pockets will govern the anisotropy in their respective mobility tensor. As a consequence, *μ* of the charge carriers in the plane perpendicular to **a** appears to be higher for these conduction channels. On the other hand, *A*_*F*_ and *m*_*eff*_ of largest Fermi pocket have been found to be 25% and 30% smaller, respectively, for *B* along (001) direction compared to the **a**-axis. So, it appears that the larger Fermi pocket has higher mobility for *B* along (001) direction compared to the **a**, unlike to that observed in two smaller Fermi pockets. However, the three-band fitting of electrical conductivity and Hall conductivity reveal that *μ* of hole-type carriers from the largest Fermi pocket is itself very small; close to one-third of the values for electron-type charge carriers from the smaller Fermi pockets, and the carrier density of hole pocket is not significantly higher than the individual electron pockets. This suggests that we can ignore the contribution of largest Fermi pocket in qualitative explanation of anisotropic transverse magnetoresistance, which will be governed by the two electron pockets of small and intermediate volume. As a consequence, the value of magnetoresistance is expected to enhance with the rotation of field from (001) to **a** direction. The three dimensional geometry of the Fermi surfaces can be constructed by observing the continuous evolution of the frequency peak associated to a particular Fermi pocket, through extensive magnetization measurements at a small angle interval between the crystallographic directions and *B*. However, such type of facility is beyond our reach at this moment.

## Conclusion

In conclusion, we have observed a large anisotropy in transverse magnetoresistance of TaSb_2_ single crystal, by rotating the field along different crystallographic directions. The large nonsaturating magnetoresistance has the maximum value ∼2 × 10^4^% and the minimum value ∼9.5 × 10^3^% at 2 K and 9 T, with the rotation of magnetic field about **b**-axis. Employing the magnetization measurement and analyzing the prominent de Haas-van Alphen oscillation, we observe three Fermi pockets. Applying field along three mutually perpendicular directions of the crystal, the cross-sectional area of the Fermi pockets has been observed to vary. Three-band fitting of electrical and Hall conductivity reveal two high mobility electron-type Fermi pockets with smaller carrier density, and a larger hole pocket with much lower carrier mobility. However, no such large difference has been found in the density of electrons and holes. This coexistence of electrons and holes of comparable density supports the electron-hole compensation mechanism as a possible origin for large and non-saturating magnetoresistance in MPn_2_ family of materials. Combining the present results of three-band analysis and quantum oscillation study, the angle dependent variation of transverse magnetoresistance in TaSb_2_ has been qualitatively explained.

## Method

### Sample preparation

Single crystals of TaSb_2_ were grown using iodine vapor transport technique in two steps. At first, polycrystalline sample is prepared by heating the stoichiometric mixture of high-purity Ta powder and Sb pieces at 650 °C for 8 h and at 750 °C for 48 h in a vacuum-sealed quartz tube. Next, the polycrystalline sample along with the required amount of iodine were sealed under vacuum in another quartz tube. Finally, the quartz tube was placed in a gradient furnace and heated for 7 days. During heating, the end of the quartz tube containing the sample was maintained at 1000 °C, while the other end was kept at 900 °C. The furnace was then cooled slowly to room temperature. Several small, shiny and niddle-like crystals formed at the cold end of the tube were mechanically extracted for transport and magnetic measurements.

### Characterization

Phase purity and the structural analysis of the samples were done by using both the high resolution transmission electron microscopy (HRTEM) in FEI, Tecnai G^2^ F30, S-Twin microscope operating at 300 kV equipped with energy dispersive x-ray spectroscopy (EDS, EDAX Inc.) unit and the powder x-ray diffraction (XRD) technique with Cu-K_*α*_ radiation in a Rigaku x-ray diffractometer (TTRAX III).

### Experimental details

The transport measurements on TaSb_2_ single crystals were done with the help of standard four-probe technique in a 9 T physical property measurement system (Quantum Design). The electrical contacts were made using silver epoxy [Epotec, USA] and thin gold wire. The magnetization was measured in a 7 T MPMS3 (Quantum Design).

### Data availability

The datasets analyzed during the current study are available from the corresponding author on reasonable request.

## Electronic supplementary material

Supplementary Information
