# Supplementary material for: Anisotropic transverse magnetoresistance and Fermi surface in TaSb2
Source: Sci Rep. 2018 Jul 12;8:10527. doi: 10.1038/s41598-018-28922-9 (PMC6043527; doi:10.1038/s41598-018-28922-9)
Supplement: Supplementary file 1 — Supplementary Information [file 41598_2018_28922_MOESM1_ESM.pdf]

Supplementary Information for "Anisotropic transverse magnetoresistance and Fermi surface in $TaSb_2$"

Arnab Pariari, Ratnadwip Singha, Shubhankar Roy, Biswarup Satpati and Prabhat Mandal
Saha Institute of Nuclear Physics, HBNI, 1/AF Bidhannagar, Kolkata 700 064, India

A. Field dependence of magnetoresistance in longitudinal configuration

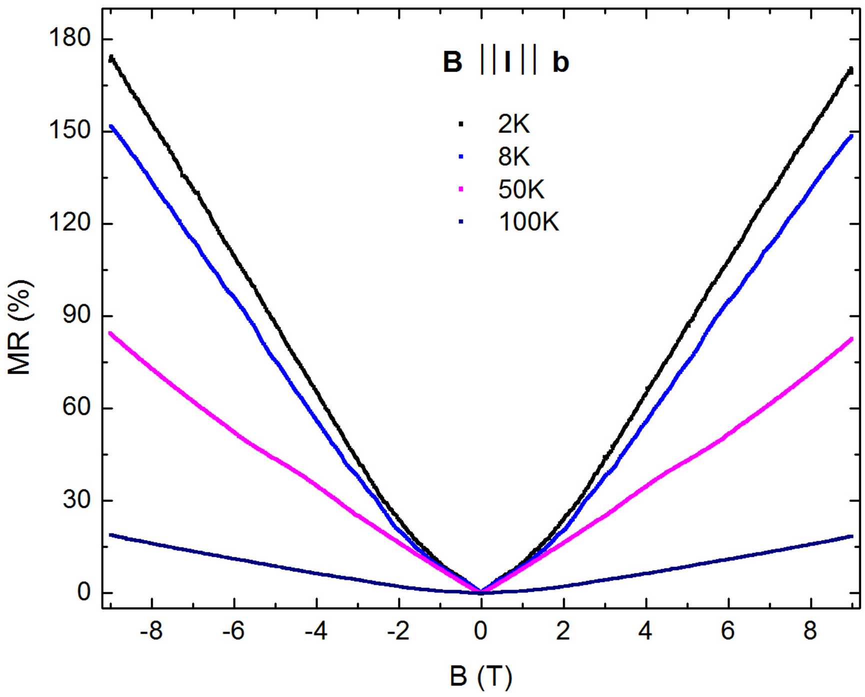

Figure S 1. Magnetoresistance as a function of magnetic field at some representative temperatures for $B \parallel I \parallel \mathbf{b}$ configuration.

We have measured the field dependence of magnetoresistance in longitudinal configuration to confirm the absence of negative LMR. Over the entire window from zero to 9 T, the LMR is positive and increases with increasing field, as shown in figure S1 at some representative temperatures from 2 to 100 K. Considering magnetoresistance $\sim$ 13000-20000 % in transverse experimental configuration, one can find that the misalignment angle between current and magnetic field is very small $\sim 0.5^\circ$ in longitudinal configuration. This unavoidable misalignment induced small positive LMR is very hard to eliminate in an experiment and frequently found in literature [S1]. Additionally, the asymmetry between the negative and positive field sections of MR is less than 3% of the total MR in longitudinal configuration. This also implies that the Hall contribution to our resistivity data is very small and contacts are good in quality.

B. Three-band analysis

Figure S2 shows the three-band fitting of electrical conductivity (σ_{xx}) and Hall conductivity (σ_{xy}) and resultant values of parameters at 100 K and 200 K. In all the fittings for different temperatures, these parameters were allowed to vary freely but their values have been found to be close to each other.

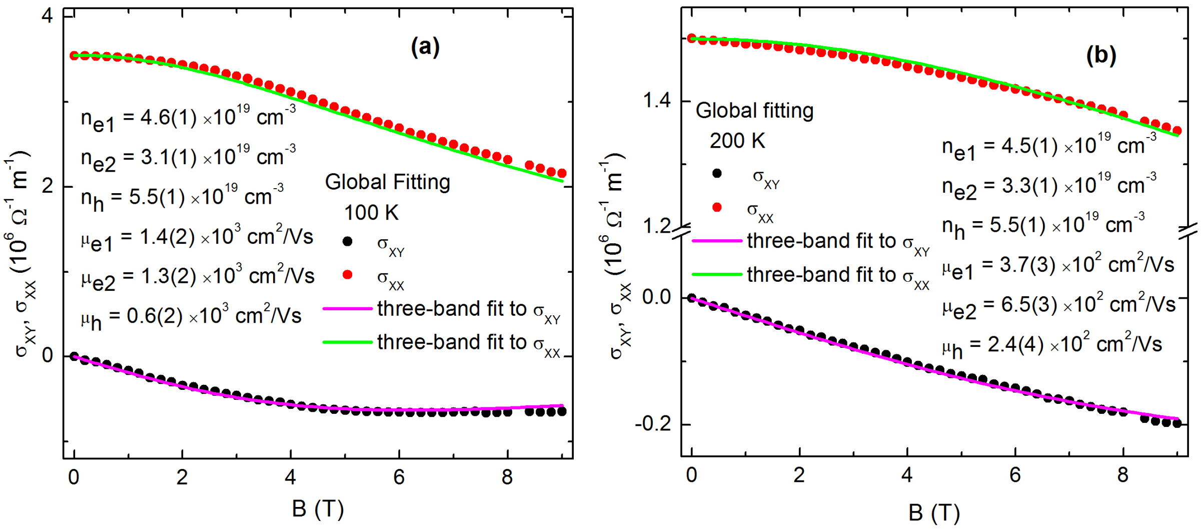

Figure S 2. (a) Global three-band fit to the electrical conductivity (σ_{xx}) and Hall conductivity (σ_{xy}) data at 100 K. The density and mobility for each types of charge carrier have been mentioned in the inset. (b) Similar three-band fit at 200 K. Inset shows the corresponding density and mobility of charge carriers.

C. Measured field dependence of magnetization (M) for all the experimental configurations.

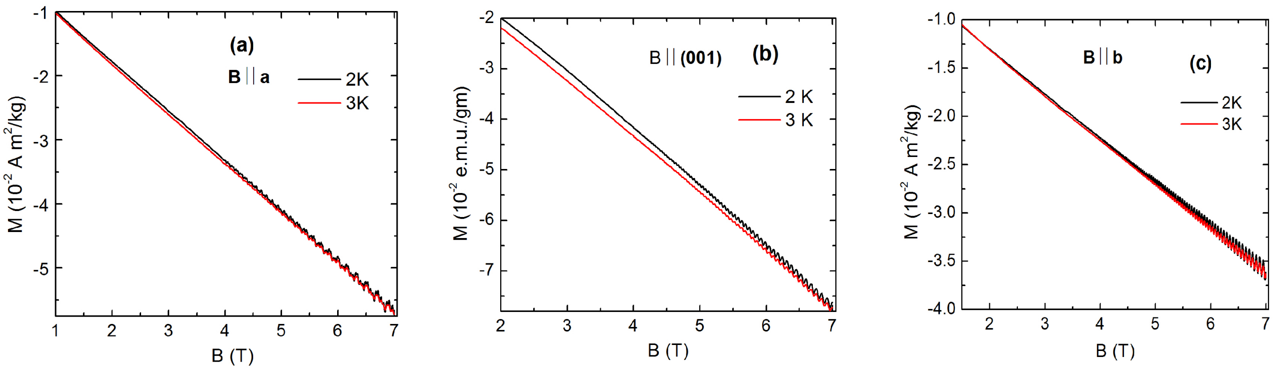

Figure S 3. Field dependence of magnetization (M) for (a) B ||**a**, (b) B ||(001), and (c) B ||**b** configurations.

D. Possible explanation for the difference in results of quantum oscillation studies on $TaSb_2$

MPn_2 class of materials show non-saturating magnetoresistance, which can be ascribed to near compensation of electrons and holes. However, in some compounds including $TaSb_2$, the electron and hole density have not been always found to be very close or completely equal to each other in spite of the fact that all the samples show non-saturating MR [S2, S3, S4, etc.]. Although, the magnetotransport experiment on TaSb2 by Wang *et al.* [S5] have reported the near perfect compensation of electrons and holes, Li *et al.* [S3] have observed electron dominated transport. Besides the experimental observations, the band structure and density functional theory calculation also show that the ratio

of electron to hole density of states at the Fermi level is 1.25:1 for $TaSb_2$, and it is not a completely compensated semimetal [S6]. This experimental and reported theoretical findings suggest that there is a redundancy in the prefect carrier compensation in $TaSb_2$, which does not affect the non-saturation field dependence of MR. If we consider a small deviation in the value of $\frac{n_e}{n_h}$ from 1, it can include or exclude the contribution of small Fermi pocket or change the volume of Fermi pockets significantly, depending on the position of Fermi level from sample to sample due to uncontrolled doping.

E. Theoretical fitting of the frequency distribution curve

To determine the intensity of the individual peaks, the Fourier transform spectra [figure 8(b) and figure 9(b)] have been fitted with the superposition of three Lorentzian distribution functions [S7], y=$\frac{a}{1+b(x-f_1)^2}+\frac{c}{1+d(x-f_2)^2}+\frac{e}{1+f(x-f_3)^2}$, where a, b, c, d ,e, f, f_1, f_2 and f_3 are the parameters. The theoretical expression fitted well with the experimental data for both the $B\|(001)$ and $B\|\mathbf{b}$ configurations. Figure S4 shows the quality of the fit for $B\|(001)$ configuration, as a representative. The parameters a, c and e, which are nothing but the actual intensity of the frequency peaks, have been determined for all the experimental temperatures. The obtained positions of the peaks (f_1, f_2 and f_3), from the fitting, have been found to be within ± 5 T of the apparent peak positions in FFT spectrum.

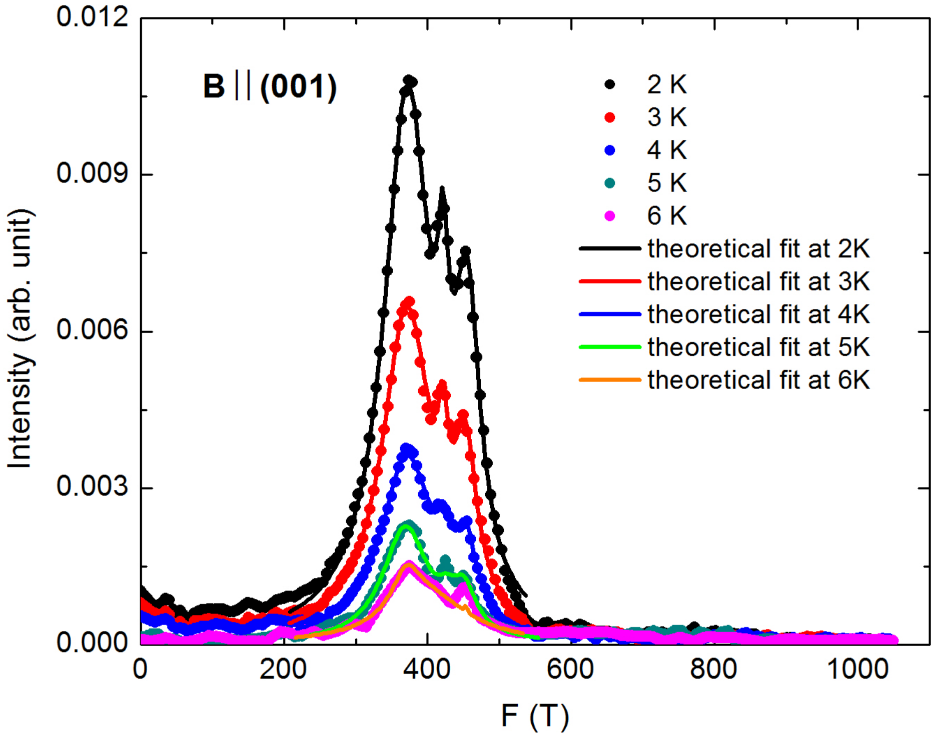

Figure S 4. Theoretical fitting of the frequency distribution plot for $B\|(001)$ configuration has been shown at different temperatures. The solid circles are the experimental data and the continuous lines are the theoretical fit with the expression, y=$\frac{a}{1+b(x-f_1)^2}+\frac{c}{1+d(x-f_2)^2}+\frac{e}{1+f(x-f_3)^2}$.

References

S1. Yuan,Z., Lu, H., Liu, Y., Wang, J. & Jia, S. Phys. Rev. B **93**, 184405 (2016).

S2. Luo, Y. *et al.* Anomalous electronic structure and magnetoresistance in $TaAs_2$. *Sci. Rep.* **6**, 27294 (2016).

S3. Li, Y. *et al.* Resistivity plateau and negative magnetoresistance in the topological semimetal $TaSb_2$. *Phys. Rev. B* **94**, 121115(R) (2016).

S4. Singha, R., Pariari, A., Gupta, G. K., Das, T. & Mandal, P. Probing the Fermi surface and magnetotransport properties of $MoAs_2$. Phys. Rev. B **97**, 155120 (2018).

S5. Wang, Z. *et al.* Topological phase transition induced extreme magnetoresistance in $TaSb_2$. Preprint at https://arxiv.org/abs/1603.01717 (2016).

S6. Xu, C., Chen, J., Zhi, G. X., Li, Y., Dai, J. & Cao, C. Electronic structures of transition metal dipnictides XPn_2 (X = Ta, Nb; Pn = P, As, Sb). *Phys. Rev. B* **93**, 195106 (2016).

S7. Shoenberg, D. *Magnetic Oscillations in Metals* (Cambridge Univ. Press, 1984).
